# Management of Mandibular Ameloblastoma: A Case Report From a Bahrain Government Hospital

**DOI:** 10.7759/cureus.104441

**Published:** 2026-02-28

**Authors:** Nadia Sikri, Sarah Eid, Sajeda Mohamed, Abhishek Ghosh

**Affiliations:** 1 Oral and Maxillofacial Surgery, Salmaniya Medical Complex, Manama, BHR

**Keywords:** extra oral approach, fibula free flap, marginal mandibulectomy, plexiform ameloblastoma, reconstruction plate

## Abstract

Ameloblastoma is a locally aggressive yet benign odontogenic neoplasm. Its three main clinical variants - intraosseous, unicystic, and extraosseous - differ vastly in their biological behavior and recurrence rate. Here we present a case of intraosseous ameloblastoma in a 32-year-old male managed by wide en bloc excision. The mandible was reinforced with reconstruction plate to prevent pathological fracture, as the residual bone height was less than 1 cm. The patient has been on regular follow-up for one year with no clinical or radiological evidence of disease. This case demonstrates that marginal mandibulectomy with reconstruction plate reinforcement can obviate the need for microvascular free fibula graft reconstruction in select cases, reducing donor-site morbidity while maintaining oncologic safety.

## Introduction

Ameloblastoma is a benign odontogenic neoplasm that is locally aggressive [1]. The lesion, if not treated properly, has a propensity for local recurrence and potential for malignant transformation [1]. The neoplasm is known to be more aggressive once it breaches its bony confines and starts interacting with surrounding soft tissue [2]. Thus, decision-making with regard to the resection margins tends to be on the more radical side. Following tumor ablation surgery, the question of reconstructing the defect arises. With modern medical advances, we have a myriad of options, including free bone grafts, microvascular free flap reconstruction in cases of segmental defects, and reinforcement with a reconstruction plate in cases of marginal mandibulectomy when the residual bone height is less than 1 cm to prevent pathological fracture. Virtual surgical planning and contemporary reconstruction strategies help us make informed decisions and should be considered when weighing marginal mandibulectomy against microvascular reconstruction [3]. Here, we present a case in which we describe intra-operative decision-making based on clinical judgment of resection margins, guided by surgical experience, clinical aggressiveness/presentation based on histopathologic variant, and patient compliance with close follow-up.

## Case presentation

A 32-year-old African male patient presented to the oral and maxillofacial surgical department after referral from the primary healthcare center with complaints of an asymptomatic swelling, slowly increasing in size over one year, on the left side of the lower jaw. There was no relevant medical history or family history. The patient was a non-smoker and non-alcoholic. On examination, he was found to have a soft to bony-hard mass along the posterior lower left gingivobuccal sulcus of the mandible in the region of canine-premolar teeth, measuring 2x3 cm in dimension. The lesion was found extending from the lower left lateral incisor to the left second premolar, identified by the bucco-cortical expansion. The lingual plate of the alveolus was found to be normal. This mass was mildly tender on palpation. All the teeth in the lower left quadrant were vital and non-tender to percussion. The overlying mucosa was normal in appearance, and the vestibular mucosa was pinchable and non-indurated. No abnormality was detected in the functioning of the mental nerve and the marginal branch of the facial nerve (CN VII). There was no palpable cervical lymphadenopathy.

**Figure 1:**
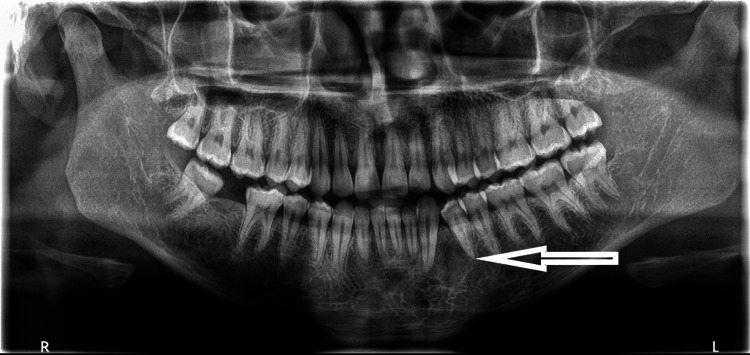
Pre-operative OPG, ill-defined multi-locular radiolucency between tooth #33 and #34, pushing the roots medially and distally, respectively, without causing root resorption. The white arrow is pointing towards the lesion. OPG: orthopantomogram

A radiographic image demonstrated an ill-defined multi-locular radiolucency involving the periapical region of the canine and first premolar teeth (Figure 1). The lesion seemed to displace the roots of the canine and first premolar medially and distally, respectively, without causing any resorption.

**Figure 2:**
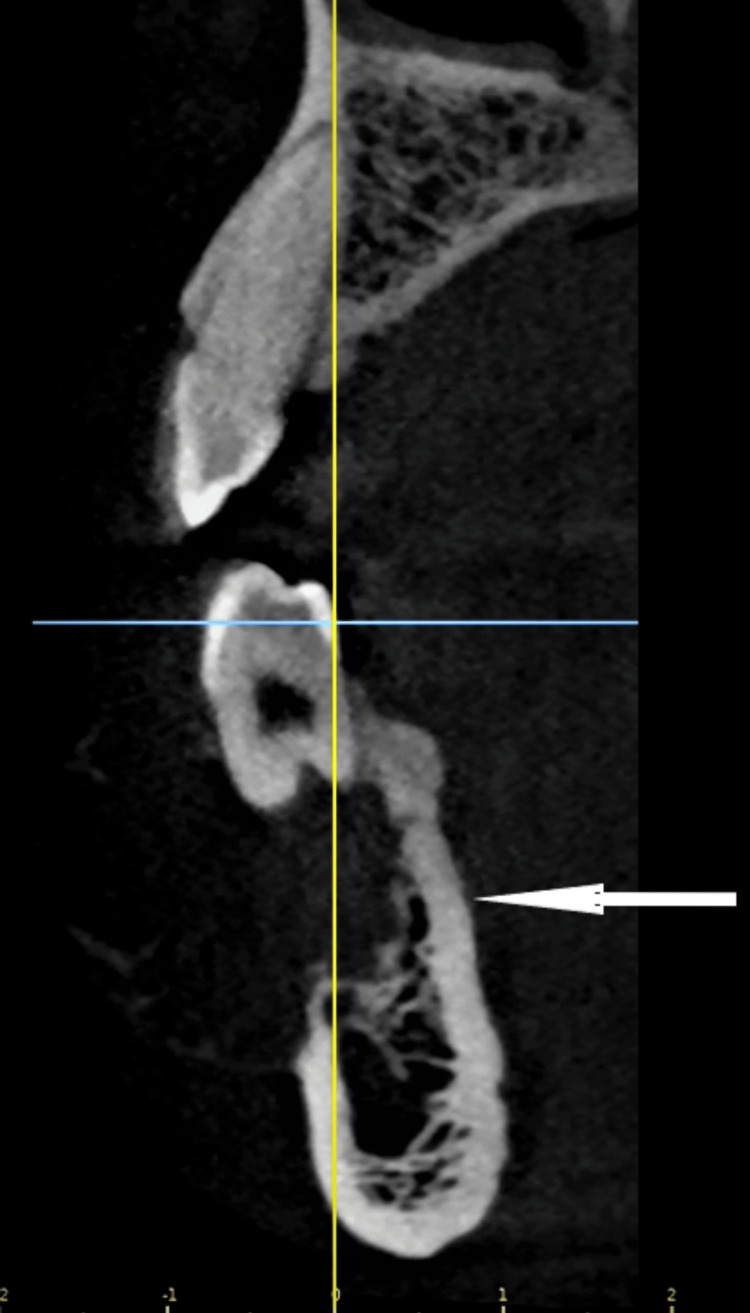
Scan section showing intact lingual plate and expanded buccal cortical plate with bony dehiscence. The white arrow is pointing towards the lesion.

Cone beam computed tomography (CBCT) imaging demonstrated that the lingual plate and mandibular inferior margin of the cortical border were intact (Figure 2). This was confirmed clinically with no "eggshell cracking" sign on palpation over the lingual cortical plate or paresthesia over the area supplied by the mental nerve. CBCT interpretation accuracy is critical for surgical planning in jaw lesions, where cross‑sectional imaging provides details about the cortical integrity [4].

Informed consent was obtained, and an incisional biopsy was performed. Clinically, no fluid content could be aspirated with a wide-bore needle. Upon histopathological examination, the lesion was found to have fibrocollagenous and bone tissue with an odontogenic epithelial neoplasm composed of islands and cords of odontogenic epithelium arranged in follicular and focal plexiform patterns in fibromyxoid connective tissue stroma. The islands exhibited peripheral palisading and stellate reticulum-like areas that were seen in between the bone trabeculae, surrounded by loose fibromyxoid stroma. No significant atypia, increased mitosis, or necrosis were noted.

Immunohistochemical studies done showed the following results. The lesion was positive for cytokeratin AE1/AE3 and CK5/6. Ki-67 index was only 2-5%, which was suggestive of the neoplasm being low grade in growth speed and aggressiveness. The lesion was negative for calretinin. Features were suggestive of an odontogenic tumor, most consistent with ameloblastoma.

The condition was discussed with the patient, and informed consent for surgical excision under general anesthesia (GA) was obtained after having explained the post-operative complications to the patient, including possible weakness of the marginal nerve and permanent anesthesia of the mental nerve.

**Figure 3:**
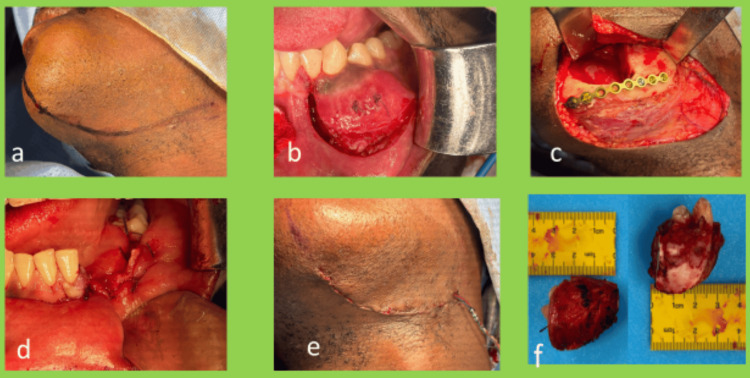
Intra-operative clinical pictures of the procedure. (a) High submandibular incision marked, (b) mucosal incision was placed with adequate margins and deepened till the bone, (c) post-resection mandibular height found to be 6 mm, so reinforced with 2 mm reconstruction plate, (d) intra-oral water-tight closure, (e) extra-oral closure in layers over suction drain, and (f) excised specimen.

The patient was taken to the OT, and GA was induced via nasal intubation. A high submandibular incision was marked and placed along the left cervical skin crease (Figure 3, panel a). An extra-oral approach was planned as the initial reconstruction plan involved multiple options (free fibula microvascular reconstruction if defect length greater than 5 cm, free iliac crest bone graft if defect length less than 5 cm, reconstruction plate reinforcement if only marginal mandibulectomy done). A subplatysmal flap was raised to the inferior border of the mandible. Marginal mandibulectomy was done from the region of tooth #32 to tooth #35 through both extra-oral and intra-oral approaches (Figure 3, panel b).

The specimen was sent for histopathologic examination and a thorough wash was given. The remaining mandibular height was less than 1 cm (approximately 6 mm). A nine-hole 2 mm thick titanium reconstruction plate was placed along with 2x8 mm titanium screws (six screws) to reinforce the mandible (Figure 3, panel c). The wound was thoroughly irrigated with saline, hemostasis was achieved, and it was closed in layers over a suction drain (Figure 3, panels d-f). The drain was removed on the second post-operative day, and the patient was discharged without any post-operative complications.

**Figure 4:**
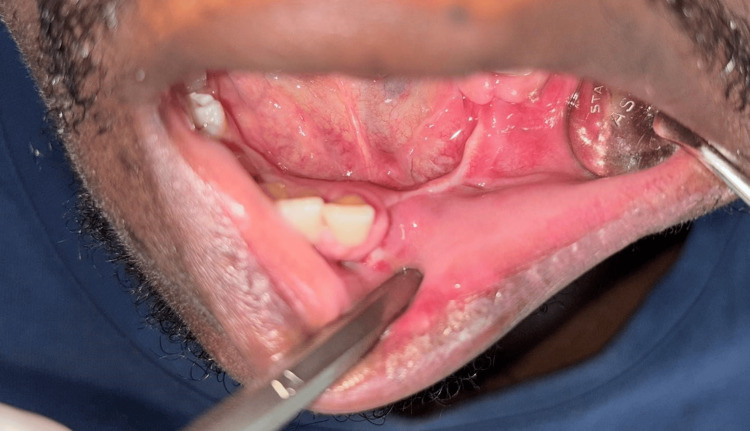
Intra-oral post-operative clinical picture at three months.

**Figure 5:**
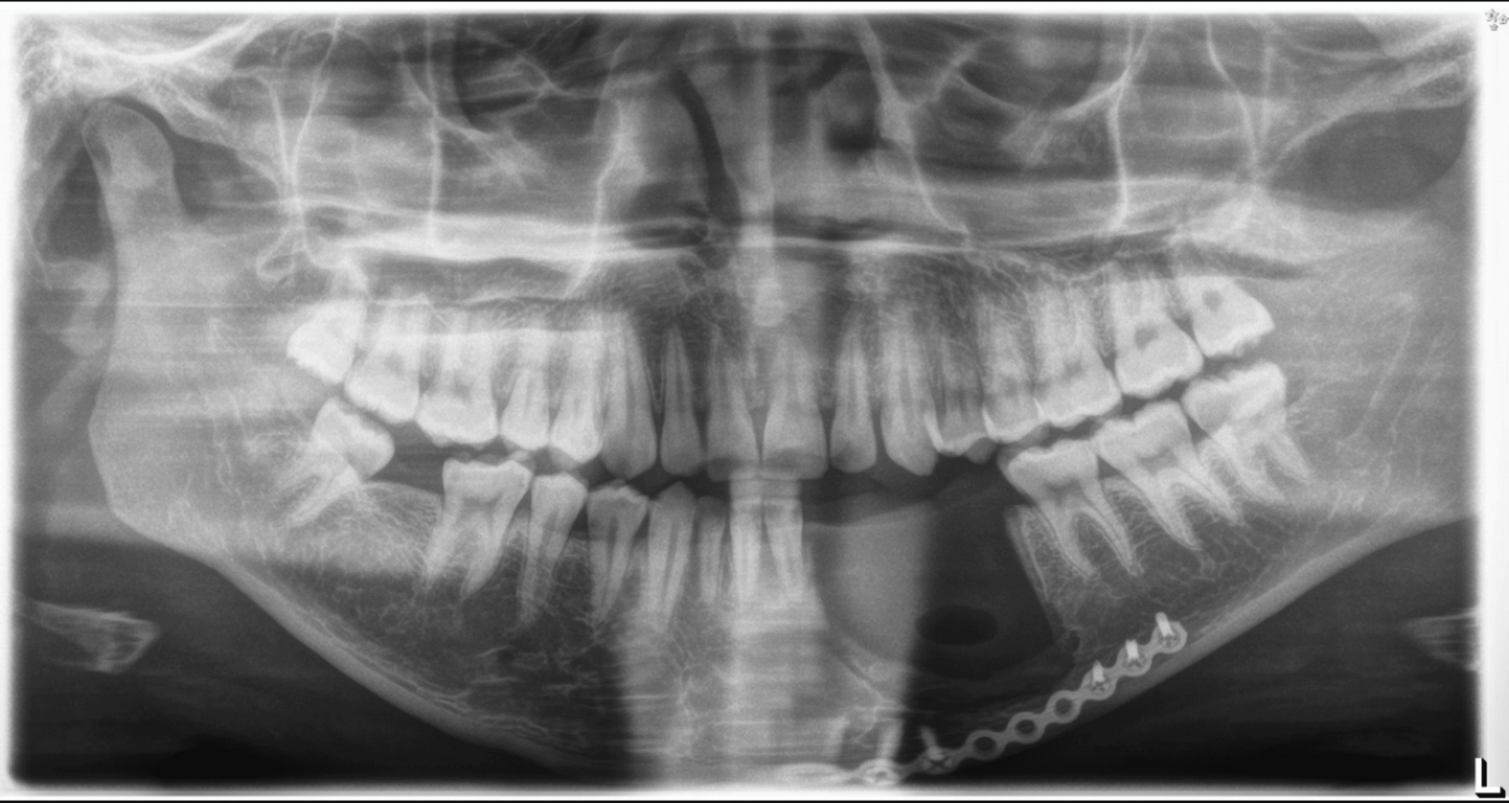
Post-operative OPG at three months. OPG: orthopantomogram

Final histopathology report showed the tumor being confined within the bone, and all resection margins to be free of tumor. No evidence of malignancy or perineural invasion was identified. Histopathological features suggested ameloblastoma. The patient has been on follow-up for the last one year with no new complaints and no evidence of disease (Figures 4, 5).

## Discussion

Ameloblastoma is a benign odontogenic tumor of the maxilla and mandible, accounting for 1% of all jaw cysts and tumors, rarely showing malignant transformation [1]. It is most commonly found between the third and fifth decades with an average age of 27 years, which is similar to the age of our patient.

The treatment modality of choice for ameloblastoma is surgery with en bloc resection [2]. High recurrence rate, particularly for the solid/multi-cystic variants (SMA), demand wide resection margins in the range of 1-1.5 cm [2]. A literature review suggests that a radical surgical approach to SMA reduces the recurrence rate; however, a recent meta-analysis could not find any conclusive evidence supporting this claim [5-7]. In our case, we chose a radical approach; however, we used negative margins rather than 1.5 cm-wide margins for the inferior border. The resultant marginal mandibulectomy defect had approximately 6 mm in height of the native mandible left. The structural integrity is compromised if the residual bone height falls below 1 cm; therefore, it was reinforced with a reconstruction plate [8]. The on-table decision to preserve the native mandible by performing marginal mandibulectomy rather than segmental was backed by the histologic variant being plexiform-follicular variant rather than desmoplastic variant (as proven in pre-operative biopsy), which is known to be more aggressive and commanding a wider margin of resection. The need for a free vascular bone graft or a microvascular bone graft was thus avoided, reducing donor-site morbidity and maintaining mandibular inferior border integrity. The patient has been on close follow-up since the surgery, and one year post-operatively has been clinically and radiologically free of disease. The need for long-term close follow-up has been explained to the patient, and the patient has been compliant.

## Conclusions

Ameloblastoma is a benign but locally aggressive enigmatic disease that requires surgical decisions custom-tailored to the clinical and histological presentation of that particular lesion. In the present case, the lesion was intraosseous without any soft-tissue breach. The lesion had a low Ki-67 index, which was favorable, suggesting a more benign lesion. Pre-operative biopsy was suggestive of a plexiform variant of ameloblastoma, which is more docile in nature. Keeping all the above characteristics of the lesion in mind, a decision was reached intra-operatively to have tumor-negative resection margins rather than a standard 1 cm free bony margin, thus preserving the natural anatomy of the mandible at the inferior border. This decision prevented donor-site morbidity, reduced operating time and costs, and resulted in faster patient discharge. The patient's compliance with a strict follow-up schedule (quarterly/every three months, clinical and annual, radiological) is also a key factor in such cases to avoid delayed presentation of possible recurrences. In our case, the patient has been compliant with regular follow-up. Thus, one has to maintain a fine surgical balance between excessively radical surgery on one hand and extreme conservative treatment on the other, keeping all the patient's demographics and lesion's characteristics in mind while deciding the surgical margins and methods of reconstruction. In conclusion, the authors would like to additionally acknowledge the inherent limitations of a case report, which preclude a blanket decision-making strategy, thereby further emphasizing the need for tailor-made approach to every lesion presenting to surgeons.
